# Epidemiology of Echinococcosis among Schoolchildren in Golog Tibetan Autonomous Prefecture, Qinghai, China

**DOI:** 10.4269/ajtmh.16-0479

**Published:** 2017-03-08

**Authors:** Huixia Cai, Yayi Guan, Xiao Ma, Liying Wang, Hu Wang, Guoming Su, Xuefei Zhang, Xiumin Han, Junying Ma, Yu Fang Liu, Jun Li, Jingxiao Zhang, Yongshun Wang, Wei Wang, Rui Du, Wen Lei, Weiping Wu

**Affiliations:** 1 National Institute of Parasitic Diseases, Chinese Center for Disease Control and Prevention, Key Laboratory of Parasite and Vector Biology, Ministry of Health, WHO Collaborating Center of Malaria, Schistosomiasis and Filariasis, Shanghai, China.; 2 Department of Parasite Control, Qinghai Province Institute for Endemic Diseases Prevention and Control, Xining, China.; 3 Endemic Disease Administration Office, Qinghai Province Health and Family Planning Commission, Xining, China.; 4 Clinical Medical Research Institute, Qinghai Provincial People's Hospital, Xining, China.; 5 Department of Science and Education, Shanghai Pulmonary Hospital, Shanghai, China.

## Abstract

Echinococcosis is a serious zoonotic parasitic disease that is highly endemic in Qinghai Province. The present study aimed to investigate the prevalence of echinococcosis among schoolchildren in Golog Tibetan Autonomous Prefecture to improve early diagnosis and treatment of patients and to provide information for echinococcosis prevention and control. A total of 11,260 schoolchildren from five counties (Maqin, Gander, Dari, Jiuzhi, and Banma) in Golog Tibetan Autonomous Prefecture, Qinghai Province, were screened for echinococcosis. Screening involved ultrasound imaging combined with serologic examination as an auxiliary diagnostic test. The prevalence of echinococcosis in the schoolchildren was 2.1% (235/11,260), with a rate of 0.8% for cystic echinococcosis (CE; 89/11,260) and 1.3% for alveolar echinococcosis (AE; 146/11,260). Additionally, one child had a mixed infection. The prevalence ranged between 1.1% and 4.1% among the five investigated counties, and was highest in Dari County (4.1%). The prevalence of echinococcosis was higher in girls than in boys and gradually increased with age. In addition, children with CE mainly had type 1 (CE1) and type 3 (CE3) lesions, and children with AE mainly had small-diameter calcified lesions, suggesting that they were in the early asymptomatic stage of echinococcosis. In conclusion, children of Golog Tibetan Autonomous Prefecture appear to exhibit the highest recorded prevalence of CE and AE globally. Ultrasound is useful for screening populations in regions where both CE and AE are endemic.

## Introduction

Echinococcosis is a serious zoonotic parasitic disease caused by cestodes. Presently, four species of *Echinococcus* are recognized as being of public health concern, namely *Echinococcus granulosus*, *Echinococcus multilocularis*, *Echinococcus oligarthrus*, and *Echinococcus vogeli*. Human cystic echinococcosis (CE) caused by *E. granulosus* and alveolar echinococcosis (AE) caused by *E. multilocularis* are the most serious forms of echinococcosis.^1^,^2^ CE is found worldwide, whereas the distribution of AE is narrower. AE is only found in cold regions in the northern hemisphere, such as Russia, Japan, China, Europe, and North America. China is reported to have the highest prevalence rates of CE and AE globally.^3^–^5^ A study conducted in 2004 reported that the prevalence of echinococcosis in 12 provinces (or autonomous prefectures) in China, based on ultrasound and serologic testing, was 1.1% and 12.0%, respectively, and that the most highly endemic areas were concentrated mainly in the northwest Tibetan region of China.^6^ Qinghai Province is one of those highly endemic areas. Surveys of echinococcosis conducted from 1995 to 2005 in different areas of Qinghai showed that the prevalence ranged between 0.2% and 8.2%, with a mean of 3.8%.^7^ The prevalence has been reported to be the highest in southeast Qinghai where mixed epidemics of CE and AE occur,^8^,^9^ making echinococcosis a serious public health problem.

Golog Tibetan Autonomous Prefecture (hereafter referred to as “Golog”) is located in the southeast of Qinghai Province, at 97°54′–120°50′ longitude and 32°31′–35°40′ latitude. The mean altitude of Golog is 4,200 m above sea level, and the annual mean temperature is −4°C. Ethnic Tibetans comprise 98% of the total population; they are primarily involved with livestock production and herding. Their lifestyle is nomadic or seminomadic. The total number of livestock in this area is > 250,000, comprising mainly yak and sheep. In addition, this area has a large number of family dogs, stray dogs, and wild canids, such as foxes and wolves. Surveys conducted between 1992 and 2007 reported the prevalence of echinococcosis to range between 4.3% and 13.7% in the six counties of Golog (Maqin, Gander, Dari, Jiuzhi, Banma, and Maduo); Gander, Dari, Jiuzhi, and Banma counties had high AE prevalence rates up to 8.2%.^7^,^8^,^10^–^12^

Echinococcosis is a chronic disease, mostly caused by parasitic infection in childhood.^2^,^13^ However, previous surveys focused on the entire population; no study has focused on echinococcosis, especially AE, among only children in Golog. The present study aimed to investigate the prevalence of echinococcosis among schoolchildren in Golog to improve early diagnosis and treatment of patients, and to provide information for echinococcosis prevention and control.

## Materials and Methods

### Ethics.

This study was approved by the National Institute of Parasitic Diseases of the Chinese Center for Disease Control and Prevention after an ethics review process. Disease screening was conducted in 2011. Prior to the survey, information regarding the purpose of the survey, methods of ultrasound and serologic detection, and provision of free treatment to those diagnosed with echinococcosis was clearly explained to the parents/guardians of the children and to the school administrative officers, and informed consent was obtained. The schoolchildren were accompanied and assisted by their head teachers during the examination.

### Participants.

**Figure 1.**
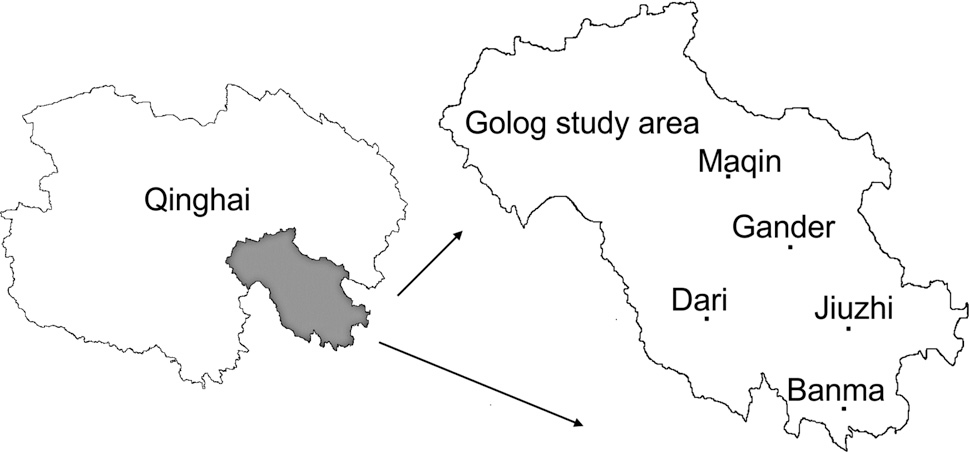
Geographic location of the areas screened in Qinghai Province, China.

According to reports of the Ministry of Education of the People's Republic of China, the net enrollment ratio into primary school is 99.7% overall and 99.8% among girls in Qinghai Province.^14^ A total of 11,260 schoolchildren from 49 elementary schools in 40 towns of five counties (Maqin, Gander, Dari, Jiuzhi, and Banma) in Golog in Qinghai were examined for echinococcosis (Figure 1

).

### Diagnostic criteria.

Abdominal scans were performed by two experienced radiologists using a Terason t2000 + Color Doppler ultrasound (3.5 Hz) system (Teratech Corp., Burlington, MA). In patients with space-occupying CE lesions, power Doppler sonography was performed to observe blood flow distribution in and around the lesions. CE and AE were classified according to the criteria recommended by the World Health Organization and the Health Industry Standards of the People's Republic of China.^3^,^15^ Based on the imaging characteristics of the lesion, CE was categorized into the following five types: CE1, CE2, CE3 (a and b), CE4, and CE5. In CE1 (unilocular hypodense cysts), the cysts are unilocular with uniform anechoic content and demonstrate pathognomonic signs including a visible cyst wall and the “snowflake” sign. In CE2 (multivesicular, multiseptated cysts with detached endocysts), the cysts are “wheel-like” structures, and the presence of daughter cysts is indicated by “rosette-like” or “honeycomb-like” structures. Daughter cysts may partly or completely fill the unilocular mother cyst. CE3 cysts include CE3a and CE3b (unilocular cysts with daughter cysts that include a solid cyst matrix or detached endocysts). These cysts demonstrate anechoic contents with detachment of the laminated membrane from the cyst wall, visible as a wavy membrane floating on top of the remaining cyst fluid (the “waterlily sign”), or as unilocular cysts that may contain daughter cysts but that are less rounded because of the decrease in intracystic fluid pressure. CE4 cysts (hyperechoic degenerative cysts) show a “ball of wool” sign indicative of degenerating membranes. CE5 cysts (heavily degenerative cysts with calcified walls) show partial to complete arch-shaped calcification, producing a cone-shaped shadow. All cases of AE have irregular or indistinct boundaries. AE is categorized into the following three types: infiltrative (heterogeneous, hyperechoic, partially calcified area), calcified (nodular, hyperechoic lesion), and central necrotic fluid (pseudo-cystic appearance due to a large area of central necrosis surrounded by an irregular hyperechogenic ring).

Serologic testing was performed on participants who had cystic lesions or in whom the diagnosis of echinococcosis was suspected, using a serologic assay detection kit for echinococcosis IgG antibody (Haitai Biological Pharmaceuticals Co., Ltd., Zhuhai, China: sensitivity 88.5% and specificity 91.7%). However, this auxiliary diagnostic test cannot differentiate between AE and CE. Participants with images typical of echinococcosis or space-occupying lesions and who tested positive for anti-hydatid antibodies were diagnosed with echinococcosis.

### Data analysis.

SPSS software (version 18.0; SPSS Inc., Chicago, IL) was used to compare the prevalence rate and other data regarding echinococcosis between the sexes, age groups, and counties. To eliminate the interference of confounding factors (such as age and sex) on comparisons of prevalence, a normalized method was used with the total number of examined persons comprising the normalized population.

## Results

### Distribution of echinococcosis in children.

Among the 11,260 schoolchildren (age range, 6–16 years) screened in this study, 235 were found to have echinococcosis. There were 89 cases of CE (38%), 146 cases of AE (61.6%), and one case of mixed CE and AE (0.4%). The total prevalence of echinococcosis was 2.1% (235/11,260), with a higher prevalence of AE than CE. The prevalence of echinococcosis was 1.7% and 2.5% in boys and girls, respectively, significantly higher in girls (Table 1).

**Figure 2.**
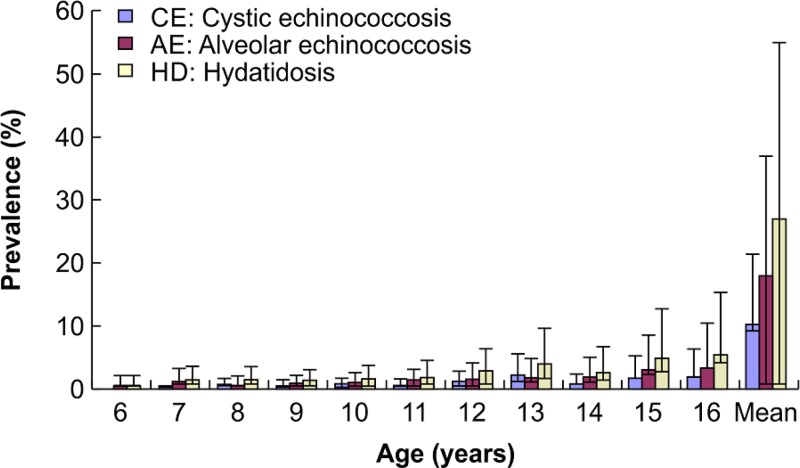
Prevalence of echinococcosis according to the age of the schoolchildren.

The mean age of the children with echinococcosis was 11.1 ± 2.2 (range, 6–16) years. The normalized prevalence in all age groups ranged from 0.5% to 5.5%. The prevalence of both CE and AE showed an increasing trend with age (Figure 2

).

In terms of the geographic differences, the prevalence of echinococcosis among the schoolchildren in the five counties of Golog ranged from 1.1% to 4.1%. Of the five counties, Dari County had the highest total normalized prevalence rate and the highest prevalence of echinococcosis, followed by Gander, Banma, Jiuzhi, and Maqin counties. The prevalence of CE was the highest in Gander County, followed by Dari, Jiuzhi, Maqin, and Banma counties. The prevalence of AE was the highest in Dari County, followed by Banma, Jiuzhi, Gander, and Maqin counties. CE and AE were found to be endemic in all five counties surveyed (Table 2).

### Distribution of different types of echinococcosis lesion.

**Figure 3.**
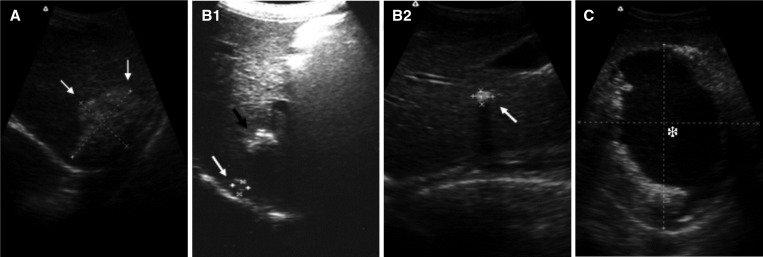
Lesions of alveolar echinococcosis. (**A**) Infiltrative lesion, (**B1** and **B2**) multiple calcified lesions and a single calcified lesion, and (**C**) central necrotic fluid.

There were 88 cases of CE, 146 cases of AE, and one case of mixed infection. Of the 88 cases of CE, 39 (44%) were CE3, 37 (42%) were CE1, eight (9%) were CE2, and four (5%) were CE4. Of the 146 cases of AE, 126 (86.3%) were the calcified type, 14 (9.6%) were the infiltrative type, and six (4.1%) were the central necrotic fluid type (Figures 3A

–D). Children with CE had up to four lesions and those with AE had up to seven lesions; however, the majority of children (69.8%, 164/235) had only one lesion (children with CE: 76.1% [67/88]; children with AE: 66.4% [97/146]). Furthermore, in all children with lesions, these lesions were found in the liver but not in other organs. The lesions were situated in the right lobe of the liver in 55.3% (130/235) of cases and in the left lobe of the liver in 23% (54/235) of cases. Both lobes of the liver were affected by one large lesion or multiple lesions in 49 cases: four had a single lesion (three children with CE and one with AE) and 45 had multiple lesions (including the child with mixed infection). In addition, one child with AE showed disease invasion into the porta hepatis.

**Figure 4.**
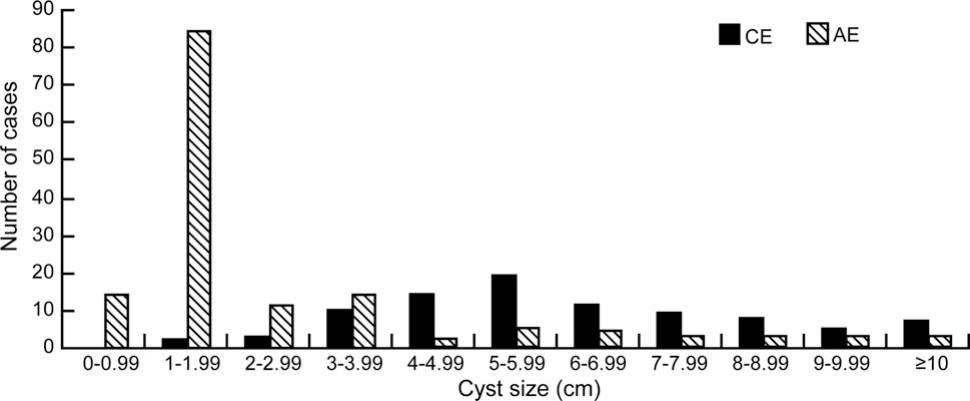
Frequency distribution of the size of the largest echinococcosis lesion in individual schoolchildren in Golog.

In children with CE, the diameter of the lesions ranged from 12.6 cm (maximum) to 1.7 cm (minimum). The mean diameter of the largest CE lesion in individual patients was 6.3 ± 2.5 cm. In patients with AE, the diameter of the largest lesion was 12.9 cm, and that of the smallest lesion was 0.97 cm. The mean diameter of the largest AE lesion in individual patients was 2.5 ± 3.9 cm. The majority of CE lesions had a diameter greater than 3 cm (94.3%, 83/88), whereas the diameter of the majority of AE lesions was smaller than 3 cm (75.4%, 110/146). The size frequency distribution is presented in Figure 4

.

## Discussion

Echinococcosis can be easily neglected,^16^ particularly in the early stage of disease. Children with echinococcosis are most representative of patients with early-stage echinococcosis. In areas where the prevalence of echinococcosis is high in the general population, the prevalence in children is usually high, too.^7^,^17^–^19^ We believe that the high prevalence of echinococcosis in western China is a threat to the health of the children in this region. The present study found that the prevalence of echinococcosis in children has reached a very serious level, 2.1%, in Golog, Qinghai. Significant geographical differences were observed in the prevalence and distribution of echinococcosis in children in the counties investigated. Schoolchildren with different severities of CE and/or AE were identified in all five counties. Dari County had the highest prevalence of echinococcosis (4.1%), and AE was the most common type. Additionally, there was one case of mixed CE and AE infection. The prevalence of echinococcosis in children from Dari County was much higher than that reported in previously published studies,^17^,^18^,^20^,^21^ indicating that Golog is highly endemic for CE and AE. Previous surveys of echinococcosis revealed that the ratios of patients with AE to those with CE were 1.48:1 in Dari County, 0.83:1 in Banma County, 0.46:1 in Jiuzhi County, and 0.41:1 in Maqin County,^8^,^11^,^12^,^22^,^23^ consistent with our findings in the children from these counties. In addition, previous animal surveys (such as those involving canids, yak, sheep, and pika) confirmed the presence and prevalence of multiple *Echinococcus* species such as *E. granulosus* (G1), *E. multilocularis*, and *E. shiquicus* in the geographic areas included in the present study.^8^,^11^,^12^,^24^–^26^ The transmission tendencies of different *Echinococcus* species might be associated with their geographical distributions, the geographical ecology, the social economy, and environmental characteristics.

Canids, particularly dogs, are the main source of infection for human echinococcosis. Previous studies have reported infections with *E. granulosus* and *E. multilocularis* among dogs in the areas included in the present study.^8^,^11^,^12^ This seems to reflect special conditions of human contamination, with dogs as an important definitive host. In addition, in the population studied, analysis of potential risk factors in all populations indicated that girls and women were at increased risk of infection compared with boys and men. Importantly, women and children have more frequent contact with infected dogs.^24^,^27^ In daily life, Tibetan women perform most of the housework. They spend most of their time around tents, where dogs and/or dog feces are present. Additionally, they take care of livestock, prepare yak dung fuel with their bare hands (the latter may be mixed with canine feces), take care of the dogs, and perform other such activities. Furthermore, Tibetan girls perform housework with their mothers or other female relatives. Therefore, exposure to *Echinococcus* eggs is more likely in girls than in boys; thus, the risk of developing echinococcosis is higher in girls than in boys. This might explain our finding that the prevalence of echinococcosis was higher in girls than in boys.

The prevalence of echinococcosis showed an overall increasing trend with age in our investigation, consistent with the characteristic long development period of most species of *Echinococcus* larvae. Since children have increased involvement in various activities with age, they have an increasing overall exposure risk with age. Moreover, the lesions increase in size with age, resulting in a higher possibility of diagnosis. This trend is consistent with the age distribution of the prevalence of echinococcosis in the entire population. In the present study, the youngest child diagnosed with echinococcosis was only 6 years old, indicating that in echinococcosis-endemic areas, children are the most representative population for early-stage echinococcosis. Owing to the special lifestyle in pastoral areas, children from these areas frequently come into direct or indirect contact with dogs infected with *Echinococcus* eggs. Moreover, poor hygiene conditions and habits, and poor economic conditions increase the chance of parasitic infection and disease. Thus, children in these areas have a high risk of infection with AE and CE.

AE progresses slowly, and the majority of patients with AE are diagnosed in adulthood. Therefore, this disease is generally diagnosed in the late stage and is difficult to treat. Pediatric patients are often neglected because they usually have small or atypical early-stage lesions. Owing to the high sensitivity of ultrasound, it can be used as a screening tool for early-stage AE in the liver.^28^,^29^ A Japanese study that focused on diagnostic imaging of AE in pediatric patients found that calcified lesions developed in only 30% of adults and in 66.6% of children with early-stage AE, indicating that the imaging characteristics of AE differ between pediatric and adult patients and that calcification might be a distinctive imaging feature of early-stage AE.^30^ In addition, an early-stage small AE lesion often contains a very small amount of fluid in the cyst and has a thick outer membrane, resulting in a strong echo on ultrasound and a calcification-like ultrasound image.^29^ In the present survey, the majority of children with AE (86%) had calcified lesions with irregular rough edges, small diameters, and scattered multiple or single high-echo areas with posterior shadows. Previous surveys in Dari and Maqin counties reported the presence of calcified lesions in 27.7% (39/141) and 38% (13/34) of adult patients with AE, respectively.^11^,^23^ Therefore, the present survey and the previous surveys in the two counties showed that the incidence rate of calcified lesions is lower in adult than in pediatric patients.

Our study found that echinococcosis lesions in children were mainly distributed in the right lobe of the liver. The majority of children with AE and CE had only a single lesion; multiple lesions were relatively rare. Among those with CE, CE1 and CE3 were the major lesion types. A small number of children had CE4 lesions, but no CE5 lesions were observed. The fact that CE4 and CE5 lesions were rare or absent indicates a short disease course in children. The finding that most children with AE had small, calcified lesions suggests that AE is at a relatively early stage in children. The lesions were larger in children with CE than in those with AE. This suggests that the growth rate of *E. granulosus* larvae is higher than that of *E. multilocularis* larvae in children. In general, the progression of AE is slow, but it might develop rapidly in some pediatric patients with AE.^31^ This was supported by our finding of central necrotic fluid in an AE lesion having a diameter of 12.9 cm in the liver of a 15-year-old boy.

A previous study reported that only 10–20% of children younger than 16 years with echinococcosis could be timely diagnosed and treated during the asymptomatic stage.^2^ In the present survey, we found that the prevalence of echinococcosis in children has reached a very serious level in Golog, and that echinococcosis was mainly diagnosed in the children in the early asymptomatic stage. The diagnosed children were provided with free drugs or were advised to undergo surgery based on the disease status; therefore, the children in this survey were diagnosed and treated in timely manner. Without echinococcosis screening, it is very difficult for such children to seek medical help for timely diagnosis and treatment. Therefore, it is important to perform echinococcosis screening of children in highly endemic areas, and to perform follow-up evaluation. In patients with calcified AE lesions, a thorough understanding of the prognosis of the lesions through follow-up observations will help in early-stage echinococcosis prevention.

In conclusion, this study has provided a more precise assessment of the prevalence of AE and CE among schoolchildren in Qinghai through census. This region appears to have the highest prevalence of echinococcosis in children in the world. Mass screening for AE and CE by means of ultrasound as a first-intention examination in economically underdeveloped regions, where there are typically poor hygienic habits and conditions, is feasible. Moreover, ultrasound examination was better accepted than serodiagnosis by the Tibetan population in the present study.

## Figures and Tables

**Table 1:** Prevalence of echinococcosis among the schoolchildren in Golog according to sex

Sex	Children surveyed (*n*)	Cystic echinococcosis				Alveolar echinococcosis				Total cases (*n*)	Total PR (%)	Total NR (%)	95% CI
		Cases (*n*)	PR (%)	NR (%)	95% CI	Cases (*n*)	PR (%)	NR (%)	95% CI				
Male	5,650	42	0.7	0.7	0.5–1.0	54	1.0	0.9	0.7–1.2	96	1.7	1.7	1.3–2.0
Female	5,610	*47	0.8	0.8	0.6–1.1	92	1.6	1.7	1.3–2.0	139	2.5	2.5	2.1–2.9
Total	11,260	89	0.8	1.6	1.3–1.8	146	1.3	2.6	2.3–2.9	235	2.1	4.2	3.8–4.5

CI = confidence interval; NR = normalized rate; PR = prevalence rate.

* Including one case of mixed infection.

**Table 2:** Prevalence of echinococcosis among schoolchildren in Golog according to county

County	Children surveyed (*n*)	Cystic echinococcosis				Alveolar echinococcosis				Total cases (*n*)	Total PR (%)	Total NR (%)	95% CI
		Cases (*n*)	PR (%)	NR (%)	95% CI	Cases (*n*)	PR (%)	NR (%)	95% CI				
Maqin	3,115	17	0.6	0.5	0.3–0.8	18	0.6	0.5	0.3–0.8	35	1.1	1.0	0.7–1.4
Gander	2,446	31	1.3	1.7	1.2–2.2	13	0.5	0.6	0.3–0.9	44	1.8	2.3	1.7–2.9
Dari	2,449	*20	0.8	0.9	0.5–1.3	80	3.3	3.5	2.8–4.3	100	4.1	4.5	3.6–5.3
Jiuzhi	1,901	18	1.0	0.9	0.5–1.3	12	0.6	0.6	0.3–0.9	30	1.6	1.5	0.9–2.0
Banma	1,349	3	0.2	0.3	0.0–0.6	23	1.7	1.7	1.0–2.4	26	1.9	2.0	1.2–2.7
Total	11,260	89	0.8	4.3	3.9–4.7	146	1.3	6.9	6.4–7.3	235	2.1	11.2	10.6–11.8

CI = confidence interval; NR = normalized rate; PR = prevalence rate.

* Including one case of mixed infection.
